# Apolipoprotein B-containing lipoproteins and atherosclerotic cardiovascular disease

**DOI:** 10.12688/f1000research.9845.1

**Published:** 2017-02-13

**Authors:** Michael D. Shapiro, Sergio Fazio

**Affiliations:** 1 Center for Preventive Cardiology, Knight Cardiovascular Institute, Oregon Health and Science University, 3181 SW Sam Jackson Park Road, Portland, OR, 97239, USA

**Keywords:** apoB, apolipoprotein B, atherosclerotic cardiovascular disease, atherogenic apoB-lipoprotein, foam cell, HDL, arterial degeneration

## Abstract

Cholesterol-rich, apolipoprotein B (apoB)-containing lipoproteins are now widely accepted as the most important causal agents of atherosclerotic cardiovascular disease. Multiple unequivocal and orthogonal lines of evidence all converge on low-density lipoprotein and related particles as being the principal actors in the genesis of atherosclerosis. Here, we review the fundamental role of atherogenic apoB-containing lipoproteins in cardiovascular disease and several other humoral and parietal factors that are required to initiate and maintain arterial degeneration. The biology of foam cells and their interactions with high-density lipoproteins, including cholesterol efflux, are also briefly reviewed.

It has been over a century since the “cholesterol hypothesis” for the pathogenesis of atherosclerosis was put forward ^1, 2^. In the ensuing decades, we learned that the key sources of cholesterol in the pathogenesis of atherosclerosis are apolipoprotein B (apoB)-lipoproteins from plasma. When one considers the totality of the evidence—from epidemiology, genetics (including Mendelian randomization studies), cell biology, experimental models, and randomized controlled clinical trials—the fundamental role of cholesterol-rich apoB-containing lipoproteins in atherosclerotic cardiovascular disease (ASCVD) is now widely held as proven, central, and causative. Low-density lipoprotein (LDL) is the principal driver of the initiation and progression of the atherosclerotic plaque ^3^. Indeed, the confirmation of a direct link between plasma cholesterol on apoB-containing lipoproteins and atherosclerosis has led to one of the greatest advances in modern medicine: the discovery and development of statins.

The fundamental role of cholesterol-rich apoB-containing lipoproteins in the genesis of atherosclerosis cannot be overstated. These atherogenic lipoproteins comprise chylomicron remnants, very-low-density lipoprotein (VLDL), intermediate-density lipoprotein, LDL, and lipoprotein(a). ApoB is a large protein that envelops the surface of atherogenic lipoproteins as a macromolecular scaffold to provide structural integrity. The apoB molecule, present in a defined stoichiometry, one single copy per particle, also serves as a ligand for LDL receptor-mediated clearance. LDL is the most abundant atherogenic lipoprotein in the fasting blood and the most prominent driver of circulating cholesterol into the artery wall. However, mounting evidence demonstrates that most apoB-containing lipoproteins (up to about 70 nm in diameter), except for fully formed chylomicrons and large VLDL, are capable of promoting plaque formation ^4^.

Although ApoB-containing lipoproteins are required for atherogenesis, they are not the only force at play, and several other humoral and parietal factors are needed to initiate and maintain the arterial degeneration process in generally reproducible and geographically confined sites within the arterial tree. These sites are non-random and are conditioned by hemodynamic parameters, such as low shear stress and non-pulsatile or non-laminar flow ^5^. These disturbances in coronary flow characteristics are related to the topography of the vascular tree and are found in areas of branching and increased vessel curvature ^6^. Although hemodynamic characteristics play an important role in the site specificity of atherosclerotic lesions, they by themselves are not responsible for the initiation of atherosclerosis. Rather, these hemodynamic factors induce specific coronary segments and their gene expression profile to differentially interact with systemic factors, resulting in susceptibility to atherosclerosis at specific locations ^7^. These local coronary hemodynamic factors and flow characteristics are intrinsically linked to endothelial function, inflammation, and the subsequent development of atherosclerosis ^5^. The low shear stress and disturbed flow play an important role in the initiation and propagation of atherosclerosis via activation of endothelial cells and upregulation of adhesion molecules on their surface. These adhesion molecules facilitate the recruitment of circulating inflammatory cells to the subendothelial space ^8^. Additionally, these same factors can alter endothelial function in a manner that impairs atheroprotective functions. Also, matrix proliferation, and hence an increased affinity for LDL retention at these sites, likely contributes to their enhanced susceptibility to atherosclerosis ^7, 9^.

As stated above, plasma apoB-containing lipoproteins penetrate the endothelial cell lining of the artery wall in susceptible regions of non-laminar flow and enter the intimal space where they may be trapped by interaction of the positively charged residues (arginine and lysine) on apoB with the negatively charged sulfate groups of subendothelial proteoglycans ^10, 11^. While LDL is trapped in the extracellular matrix, LDL receptors (LDLRs) on foam cells can recognize native or minimally modified LDL (MM-LDL), oxidized LDL without extensive protein modification ^12^. While apoB-containing lipoprotein retention within the arterial wall is initially related to direct binding of LDL to proteoglycan glycosaminoglycan chains, infiltration of the intima by macrophages that secrete bridging molecules, such as lipoprotein lipase, triggers a transition to indirect binding of apoB-containing lipoproteins. These bridging molecules work together in sync with other proatherogenic modifications of the extracellular matrix and LDL, culminating in enhanced retention of atherogenic lipoproteins ^13^. As the oxidation of the lipoprotein becomes more profound, its affinity for the LDLR diminishes, but its ability to get inside cells actually increases because of the action of scavenger receptors such as scavenger receptor-A (SRA) and CD36 ^14^. Unlike the LDLR, scavenger receptors are not subject to feedback regulation by cellular cholesterol levels; thus, arterial macrophages can internalize unregulated quantities of cholesterol ester and eventually transform into foam cells ^15, 16^. This lack of feedback regulation elevates the quantitative importance of the scavenger receptor above that of LDLR in terms of the amount of cholesterol uptake by arterial macrophages. Interestingly, triglyceride-rich apoB-containing lipoproteins (that is, remnants) do not require oxidative modification to be recognized and massively taken up by arterial macrophages. Furthermore, these remnant lipoproteins incite a more profound inflammatory response than do LDLs ^17^. The debate regarding the relative atherogenic potential of LDL versus other apoB-containing lipoproteins rages on and remains unresolved. However, one must keep in mind that, possibly with the exception of severe familial hypercholesterolemia, the etiology of atherogenesis in the typical person reflects more the accumulation of remnant lipoproteins than that of pure, triglyceride-depleted LDL. This is known as the post-prandial hypothesis of atherogenesis, first formulated nearly 70 years ago ^18– 20^.

Although most attention has been focused on the role of oxidized LDL in foam cell formation, it is also important to consider that non-oxidized, modified forms of LDL (small dense, electronegative, and especially desialylated) have been implicated in atherogenesis as well ^21^.

Cholesterol-laden foam cells activate a gene expression program that augments inflammatory pathways and induces production of various proteases (for example, collagenases, elastases, and cathepsins) ^22^. Cumulatively, this has the effect of recruiting more monocytes into the coronary intima and of opening up passages for the arrival of smooth muscle cells from the media ^23^. The current view of this process sees the initial response to the subendothelial retention of lipoproteins as an appropriate and measured attempt to clear unwanted and dangerous debris from the artery wall. Ultimately, however, the ensuing chronic inflammatory response becomes maladaptive in advanced atherosclerosis largely due to altered behavior of arterial phagocytes which underlie defects in inflammation resolution ^24^. Owing to the lipid load, vascular foam cells lose the mobility typical of inflammatory cells and are unable to egress out of the arterial wall. In addition, during the early stages of plaque development, apoptotic cells are taken up by other phagocytes in a process called efferocytosis and are effectively cleared. However, late-stage atherosclerosis is characterized by defective efferocytosis which leads to an increased inflammatory response, necrotic core expansion, and plaque progression. Macrophage necrosis leads to an even more prominent inflammatory response in a self-perpetuating cycle.

As discussed thus far, apoB-containing lipoproteins are intrinsically linked to the initiation, development, and propagation of atherosclerosis. On the other hand, high-density lipoprotein (HDL) is seen as anti-atherogenic because of its role in cellular cholesterol extraction and reverse cholesterol transport. Animal experiments involving transplantation of atherosclerotic aortic segments into normolipidemic hosts demonstrate decreases in the macrophage content of the transplanted aorta ^25^. Furthermore, this response is exaggerated by overexpression of apolipoprotein A1 (apoA-I) in the recipient ^26^. However, new insights into HDL biology are yielding a more complex story. Although targeting LDL cholesterol (LDL-C) has had stunning results, it is distressing that interventions targeting HDL cholesterol (HDL-C) have not yielded benefit, given that the epidemiological association of HDL-C and ASCVD is at least as strong as that of LDL-C ^27– 29^. As it turns out, HDL-C, a static measure of cellular cholesterol carried by plasma HDL, may be a poor surrogate for the key biological activities of HDL. Although HDL performs myriad non-redundant functions that extend beyond lipid metabolism (for example, anti-oxidative, anti-platelet, anti-inflammatory, and anti-apoptotic properties), its role in reverse cholesterol transport may be its most important with regard to mitigating plaque development, vulnerability, and (ultimately) catastrophic atherosclerotic events ( Figure 1) ^30^. In that regard, a dynamic measure of HDL function may enhance its prognostic capability. An initial investigation revealed that assaying cholesterol efflux from cultured cells (the first step in reverse cholesterol transport) was more closely correlated with carotid intima media thickness and angiographic coronary artery disease when compared with HDL-C ^31^. Another study demonstrated that cholesterol efflux capacity predicts incident ASCVD events ^32^. These findings have been validated in an additional large study ^33, 34^ but challenged in another ^34^. HDL consists of particles that vary in size, composition, and function ^35^. Presumably, at least some of the functional heterogeneity of the HDL spectrum is explained by differences in its proteome and lipidome ^36, 37^. This facet of HDL biology is a current focus of intense investigation that may bear the fruit of more intelligent drug development.

**Figure 1.**
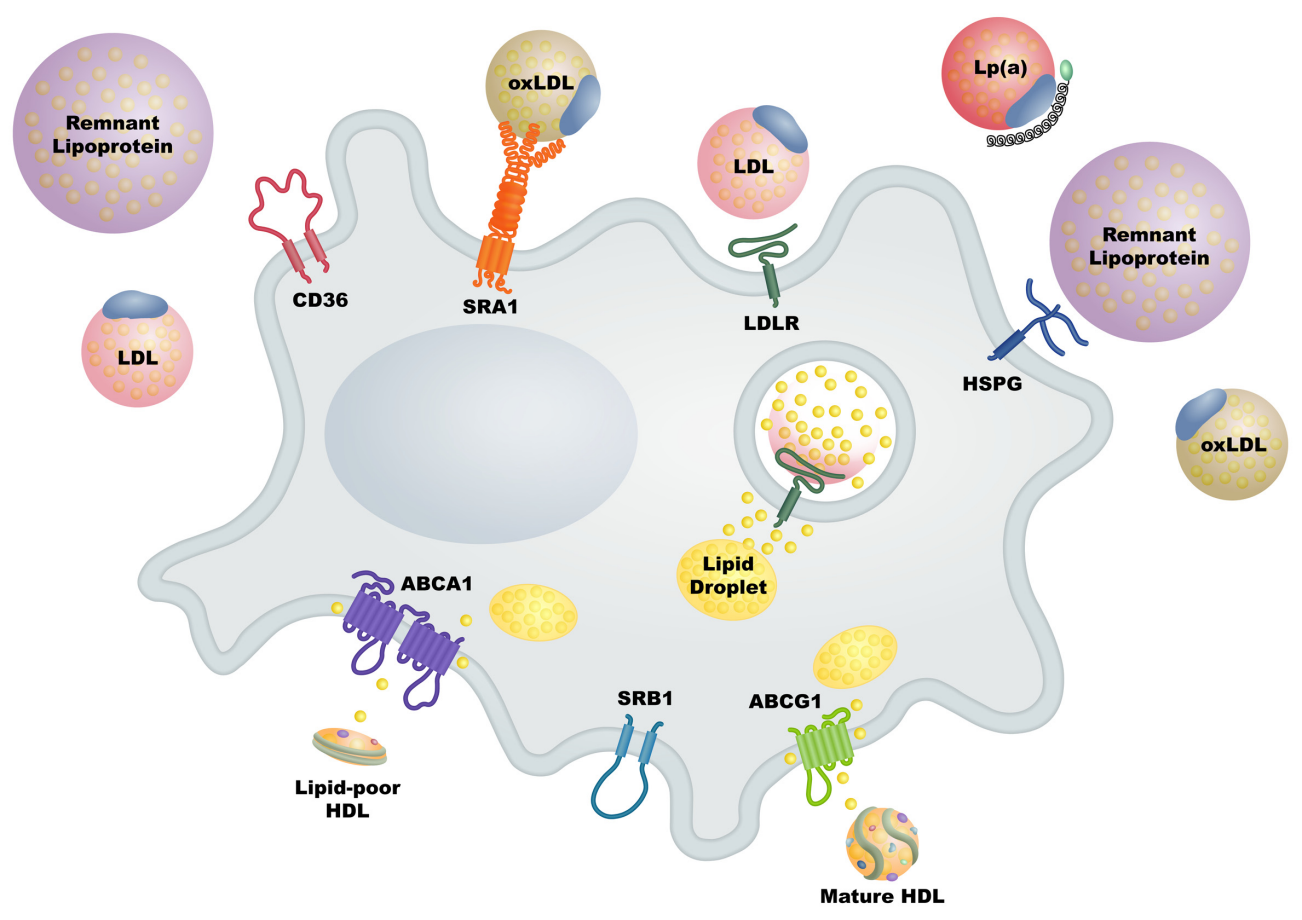
The odds are stacked against atherosclerotic plaque regression. The arterial wall is under constant assault by a variety of atherogenic particles, each carrying a large cholesterol cargo. While a foam cell takes up hundreds of molecules of cholesterol from each atherogenic particle via a wide array of receptors, it can only eliminate cholesterol through channels that allow the passage of few molecules at a time. ABCA1, ATP-binding cassette transporter A1; ABCG1, ATP-binding cassette transporter G1; HDL, high-density lipoprotein; HSPG, heparin sulfate proteoglycans; LDL, low-density lipoprotein; LDLR, low-density lipoprotein receptor; Lp(a), lipoprotein(a); oxLDL, oxidized low-density lipoprotein; SRA1, scavenger receptor A1; SRB1, scavenger receptor B1.

The necrotic core is not the only compositional change affecting plaque size and stability. Advanced plaques are also marked by the presence of cholesterol crystals. Interestingly, some of the crystals are derived from erythrocytes, whose membranes are the richest in free cholesterol among all cells in the body. Intraplaque hemorrhage has emerged as a significant contributing factor to enlargement of the necrotic core ^38^. The source of hemorrhage is thought to arise from leaky new capillaries that infiltrate the plaque as futile neovascularization attempts in response to a hypoxic environment created by increased lesion burden and inflammatory macrophages ^39^. The capillaries within the plaque typically lack an intact basement membrane, are poorly stabilized by surrounding pericytes, and show less than tight endothelial junctions, all factors likely responsible for their inability to hold contents.

Macrophage engulfment of cholesterol crystals or *de novo* formation of intracellular cholesterol crystals will induce lysosomal destabilization and release of cathepsin B to the cytoplasm, which activates a multimolecular signaling complex known as the nucleotide-binding leucine-rich repeat-containing pyrin receptor 3 (NLRP3) inflammasome ^40^. Activation of the NLRP3 inflammasome results in caspase-1-mediated production of interleukin-1 beta (IL-1β) and ultimately IL-6, which amplifies the inflammatory cascade ^41^. The significance of this discovery needs to be stressed, as it offers a mechanistic relationship between hypercholesterolemia and vascular inflammation ^42^. The importance of cholesterol crystals within foam cells extends beyond its ability to augment inflammation. Crystalline cholesterol may also provoke plaque rupture by physical disruption of the fibrous cap ^43^.

Abela and Aziz ^44, 45^ and Kellner-Weibel *et al*. ^44, 45^ investigated the role of crystalline cholesterol in advanced atherosclerotic lesions. They observed that crystallization of cholesterol can result in sharp-edged cholesterol crystals with the potential to penetrate biological membranes. They hypothesized that these cholesterol crystals could induce plaque rupture by mechanical perforation of the outer layers of atherosclerotic plaques. To support this hypothesis, they used scanning electron microscopy to demonstrate cholesterol crystals perforating the arterial intima in patients who had died from acute coronary syndromes ^46^. The authors found no cases of cholesterol crystal perforation in subjects with severe atherosclerosis but without acute cardiac events. These pioneering studies were the first to suggest that cholesterol crystals can trigger plaque disruption and vascular injury. However, although these studies are compelling, it is not entirely clear whether cholesterol crystals are causally linked, or are merely bystanders, to plaque rupture.

The focus of this review has been on experimental models of atherosclerosis spanning numerous decades. However, several orthogonal lines of evidence have now clearly established the link between lipids and ASCVD. Starting with the visionary Framingham Heart Study in 1948, numerous large epidemiological studies performed around the globe provided highly reproducible results ^47– 51^. The consistency of the epidemiology was truly stunning and suggested the association of LDL-C with ASCVD. Demonstration of the causal role of LDL with ASCVD emerged from genetics (familial hypercholesterolemia, genome-wide association studies, and Mendelian randomization studies). Individuals with genetically elevated LDL-C are at high risk for ASCVD, whereas individuals with genetically low LDL-C are at exquisitely low risk for ASCVD. The results of the large prospective, double-blind, randomized, placebo-controlled statin mega-trials further supported the notion that LDL is causal in ASCVD, although many investigators for years attributed the benefits of statins to their “pleiotropic” effects ^52– 57^. The results of the IMPROVE-IT trial (Improved Reduction of Outcomes: Vytorin Efficacy International Trial) finally created a wedge between certainty and doubt ^58^. All things considered, there is now unequivocal evidence that cholesterol-rich apoB-containing lipoproteins are inextricably linked with ASCVD and are the principal drivers of this process. For two and half decades, statins enjoyed a privileged status; they were considered the most effective class of drugs to reduce LDL-C and ASCVD events to which no additional drug impacted outcomes. The IMPROVE-IT trial ushered in the new era where LDL lowering with non-statin agents has demonstrated the ability to add to the benefits of statin therapy ^58^. This has brought in renewed energy for the discovery of novel cholesterol-lowering strategies. Within the last decade, investigators have successfully linked genetic insights to molecular pathways, allowing the swift development of a new class of potent LDL-C-lowering drugs, the proprotein convertase subtilisin-kexin type 9 (PCSK9) inhibitors ^59– 61^. These agents hold the potential to transform ASCVD risk reduction given their tremendous LDL-lowering power ^62^. However, is it likely that the epidemic of cardiovascular disease will be stopped by an LDL-lowering agent usually started when the patient is near to, or has already had, the first ischemic event? We think not. The real revolution in the prevention and management of ASCVD will arrive with tools that prohibit plaque development (needed by large numbers of relatively young and healthy individuals) and tools that induce plaque regression (needed by patients with established disease). These tools are likely to affect parietal processes, such as endothelial function, inflammatory responses, macrophage survival and egress, and lipid efflux. At last check, nothing in the literature forecasts the arrival of these tools in practice anytime soon.
